# Reply to Henehan and Witts: Continental flood basalts drive extinctions after the mid-Mesozoic

**DOI:** 10.1073/pnas.2304194120

**Published:** 2023-05-15

**Authors:** Theodore Green, Paul R. Renne, C. Brenhin Keller

**Affiliations:** ^a^Department of Earth Sciences, Dartmouth College, Hanover, NH 03755; ^b^Department of Geosciences, Princeton University, Princeton, NJ 08544; ^c^Berkeley Geochronology Center, Berkeley, CA 94709; ^d^Department of Earth and Planetary Science, University of California, Berkeley, CA 94720

The temporal coincidence of large igneous provinces (LIPs) with Phanerozoic extinctions (e.g., ref. 1) has encouraged investigations of causality. Green et al. (2) found a greater temporal correlation between LIPs and Phanerozoic extinctions than expected from random chance. Henehan and Witts (3) suggest that, though earlier LIPs may have caused extinctions, the mid-Mesozoic rise of pelagic marine calcifiers prevented later volcanism like the Deccan Traps from significantly perturbing the ecosystem. Here, we address this comment (3) by calculating the coincidence product (2) with a timeframe restricted to the mid-Mesozoic through Quaternary.

**Fig. 1.**
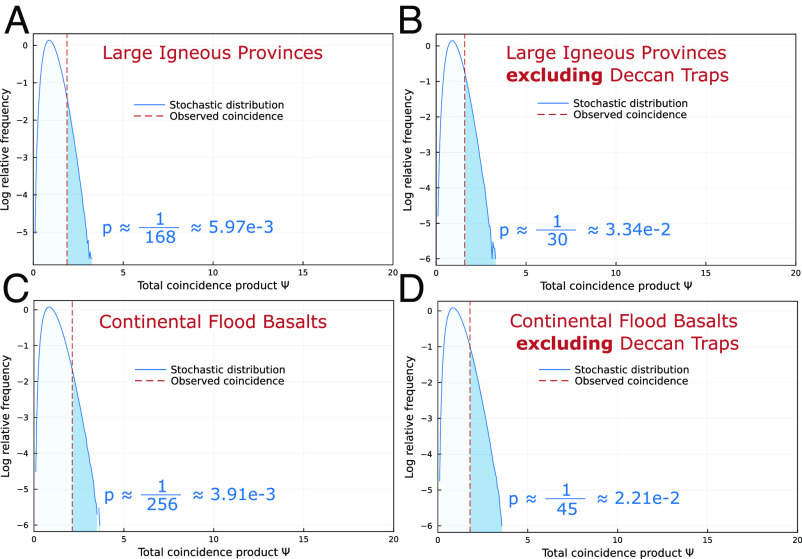
Relationship between observed and expected coincidence products, mid-Mesozoic to present. The observed coincidence products between stage boundaries from the mid-Mesozoic (190.8 Ma, base-Pliensbachian) to the Quaternary and all large igneous provinces in that interval (*A*), along with the corresponding stochastic distribution (which would result if timescale boundaries were spread randomly throughout that interval following a uniform distribution), on a logarithmic *y* scale. *P* gives the probability that a uniform distribution has a higher coincidence product (*Ψ*, ref. 2) than observed. (*B*) shows the results for LIPs and stage boundaries when the Deccan Traps, the largest LIP correlated with a severe extinction in that interval, is excluded. (*C*) shows the coincidence products for only the continental LIPs, called continental flood basalts, and the stage boundary extinctions in this interval, while (*D*) shows that relationship with the Deccan Traps excluded. There is a statistically significant relationship between LIPs and extinctions after the mid-Mesozoic, even when the influence of the Deccan Traps is excluded. The difference in timeframe and the number of events considered mean that these values should not be directly compared with those calculated for the whole Phanerozoic (2), though the results are statistically significant for both intervals.

To examine the correlation between LIPs and extinctions after this change in biogeochemical conditions, we consider only stage boundaries and LIPs occurring in the last ∼190 Myr, beginning shortly after the initial rise of pelagic marine calcifiers and allowing for ∼10 Myr of ecosystem recovery after the Triassic–Jurassic mass extinction (4). Fig. 1 shows that there is a significant correlation between LIPs and stage boundary extinctions even when excluding the Paleozoic and early Mesozoic. The relationship remains greater than expected from random chance when the Deccan Traps are excluded. The CaCO_3_ saturation state of the oceans (e.g., ref. 5) may contribute to the decreased severity of more recent extinctions, but these events maintain the potential to dangerously perturb the Earth system given a sufficient carbon input rate (6), though high-eruptive-rate LIPs are rare in this interval. Therefore, the significant relationship between LIPs and extinctions in this interval warrants consideration.

As discussed in Green et al. (2), the greater-than-chance temporal coincidence between LIPs and extinctions does not establish causality for any single extinction. That determination would require detailed study of the Earth system during an event rather than an evaluation of the correlation across multiple intervals presented here. At the much-discussed Cretaceous–Paleogene mass extinction, for example, assessments of causal relationships would need to examine all available data, including those suggesting prolonged stress leading to gradual extinction before the boundary (e.g., ref. 7) and those favoring abrupt extinction at the boundary (e.g., ref. 8). Also necessary to consider would be the relationship of individual eruptive pulses and volatile emissions to climate perturbations like the Late Maastrichtian Warming Event (e.g., refs. 9, 10, and 11). However, it is noteworthy that the Cretaceous–Paleogene boundary is a severe extinction temporally associated with a high-eruptive-rate LIP, which fits with the broad LIP–extinction correlations discussed by Green et al. (2). Though it does not determine causality for individual events, the temporal coincidence of LIPs and extinctions across the Phanerozoic demands an explanation. Absent any known mechanism to generate both LIP-creating mantle plumes and, independently, biotic extinctions (and discounting the possibility of extinctions causing LIPs), we are left with either a causal relationship between LIPs and extinctions or random acausal chance. Since Green et al. (2) and this present correspondence quantify the latter, finding the correlation between LIPs and extinctions in both cases unlikely to result from random chance, it is reasonable to explore LIPs as a causal mechanism for Phanerozoic extinctions even after the mid-Mesozoic.
